# Structural Features of Root Cells of *Nicotiana tabacum* Grown in the Presence of Short Peptides AEDL and Its Methylated Analog AED(OMe)L

**DOI:** 10.3390/ijms27156768

**Published:** 2026-07-28

**Authors:** Elena Michailovna Lazareva, Eugeniy Pavlovich Kazakov, Neonila Vasilievna Kononenko, Larisa Ivanovna Fedoreyeva

**Affiliations:** 1 All-Russia Research Institute of Agricultural Biotechnology, Timiryazevskaya 42, Moscow 127550, Russia; lazareva-e@yandex.ru (E.M.L.); nilava@mail.ru (N.V.K.); 2 Biological Department, M.V. Lomonosov Moscow State University, Leninskie Gory 1, Moscow 119991, Russia; 3 A.N. Belozersky Research Institute of Physicochemical Biology, Lomonosov Moscow State University, Leninskie Gory, 1, building 40, Moscow 119991, Russia

**Keywords:** short peptides, TOR kinase, ATG proteins, valuoles, autophagosomes, plastids, *Nicotiana tabacum*

## Abstract

Peptides as signaling molecules play an important role in intercellular communication. Exogenous peptides AlaGluAspLeu (AEDL) and AlaGluAsp(OMe)Leu (AED(OMe)L) at a concentration of 10^−7^ M stimulate the growth and development of *Nicotiana tabacum*. A detailed study of *Nicotiana tabacum* root cells grown in the presence of exogenous peptides AEDL and AED(OMe)L using transmission electron microscopy revealed characteristic differences in the ultrastructure of some cytoplasmic organelles compared to control cells. Importantly, vacuoles and autophagosomes differing in size and content, as well as amyloplasts and proteinoplasts never previously described in the literature, were found in the cells of the outer and inner root cortex. Only lytic vacuoles were detected in the cytoplasm of control cells, whereas in the presence of peptides, predominantly protein-storing vacuoles were found in root cells. In the presence of the exogenous short peptide AEDL, tobacco root cells contained amyloplasts with numerous large starch granules in the stroma, which were not detected in control cells. In the presence of the modified short peptide AED(OMe)L, leukoplasts contained protein bodies. Moreover, in contrast to control cells and cells grown in the presence of the AEDL peptide, a megaphagic (pexophagic) variant of autophagosomes with peroxisomes was detected for the first time in tobacco cells treated with AED(OMe)L. Characteristic types of phagophores were identified, forming numerous small autophagosomes with cytoplasmic regions, multivesicular bodies or concentric membranes, and cytoskeletal elements. Data on the expression of the *ATG*, *TOR*, and *FREE1* genes confirmed the pattern of the existence of a large number of small autophagosomes. Based on the obtained data, a scheme for the regulation of the formation of root architecture of *Nicotiana tabacum* in the presence of AEDL and AED(OMe)L was proposed.

## 1. Introduction

Storage proteins are deposited in PSVs, which are formed from pre-existing embryonic vacuoles [1]. As plants develop, PSVs are transformed into acidic degradation compartments filled with active proteases, becoming LVs, which degrade storage proteins, lipids, and starch for use as building blocks [2]. The plant protein FREE1, which contains the FYVE (endosomal sorting) domain, is a member of the ESCRT complex. Mutant plants lacking a functional FREE1 gene have impaired multivesicular body (MVB) biogenesis and vacuolar membrane protein sorting [3]. Loss of FREE1 function, in *free1* mutants, results in severe impairments of vacuolar protein transport, vacuole biogenesis, and autophagosome degradation in vacuoles [4,5]. *Free1* mutants have been shown to accumulate a high number of autophagosomes [6]. Autophagosomes are morphologically distinct from multivesicular bodies [7]. A number of conserved proteins (ATG) are associated with the autophagy process [8]. Free1 mutants were found to have significantly increased levels of lipidated ATG8 conjugated with phosphatidylethanolamine (PE), which marks nascent/completed autophagosomes [9]. The accumulation of autophagosomes and a significant increase in ATG8-PE likely indicate defects in membrane fusion between endosomes or autophagosomes and vacuoles, but not the induction of autophagy due to FREE1 deficiency [9]. Yeast two-hybrid analysis showed that FREE1 does not directly interact with ATG8 proteins, but interacts with them indirectly through the phosphatidylinositol 3-kinase-like kinase (PI3K), thereby regulating autophagosome formation [9].

A large class of peptide hormones is involved in a variety of cellular functions and is essential for cell differentiation, as well as plant growth and development [10,11,12,13]. Peptides are an important class of biomolecules for intercellular communication and signaling. Some of these peptides act as local signals during plant growth and development, while others are long-range signals from roots to shoots, necessary for adaptation to the environment. Peptides have been detected in all plant organs. Depending on their localization, peptides possess specific functional activity.

Previously, we observed stimulation of *Nicotiana tabacum* development in the presence of the acidic tetrapeptide AlaGluAspLeu (AEDL) [14]. The localization of ATG8 on the surface of autophagosomes was studied in control and peptide-treated conditions. For the first time, the ultrastructure of cells in the inner and outer cortex of tobacco roots was studied, the cells of which had their own characteristics. An increase in the number of small autophagosomes occupying cytoplasmic regions near cell membranes was detected. However, their number did not exceed the basal level of autophagosomes in control cells, which is necessary to maintain homeostasis. Vacuoles in control cells were lytic, while those in cells grown in the presence of AEDL were PSVs. Different types of phagophores and autophagosomes formed under the influence of AEDL were represented. Changes in the structure of leukoplasts in cells of the outer cortex attracted particular attention. Amyloplasts with numerous starch grains appeared in cells of the outer cortex, while most leukoplasts in the control and inner cortex cells did not accumulate starch. This indicated that carbohydrate metabolism in the outer layers of root cells was accelerated in the presence of AEDL, and excess starch was stored in the stroma of leukoplasts [14].

Previously, we observed stimulation of *Nicotiana tabacum* development in the presence of the acidic tetrapeptide AEDL [14]. It was suggested that AEDL is involved in the regulation of cell proliferation and differentiation [15]. It is known that to regulate the processes of proliferation and differentiation of stem cells, a redox balance is required, the main participants of which are H_2_O_2_ and O_2_^•−^ [16]. It has been proven that the redox balance determines the fate of stem cells [16]. Regulation of the redox balance occurs through a negative feedback mechanism. O_2_^•−^ is localized on the periphery of the stem cell zone and is involved in the conversion of O_2_^•−^ to H_2_O_2_.

In connection with the above, it is interesting to study the influence of the magnitude of the negative charge of the AEDL peptide on the development of *Nicotiana tabacum*. For this purpose, the methylated peptide AED(OMe)L was synthesized. The purpose of this study was to comparatively study the structural features of *Nicotiana tabacum* root cells grown in the presence of the exogenous short peptide AEDL and its methylated analogue AED(OMe)L.

## 2. Results

### 2.1. Morphometric Parameters

We previously demonstrated that the AEDL peptide activates *Nicotiana tabacum* development [14]. Tobacco grown in the presence of 10^−7^ M AEDL exhibited larger shoots and root systems compared to the control (Figure 1, Table 1). Reducing the negative charge of the AED(OMe)L peptide by methylating the carboxyl group of aspartic acid further activates the growth of *Nicotiana tabacum* stems and leaves. Interestingly, the root systems of *Nicotiana tabacum* grown in the presence of AEDL and AED(OMe)L had a more robust architecture compared to the control. Moreover, the above-ground portion of plants grown in the presence of AED(OMe)L was larger than in the control or in the presence of AEDL. It is possible that the magnitude of the negative charge of the exogenous peptide influences the architecture of *Nicotiana tabacum* plants.

### 2.2. Analysis Ultrastructure of the Roots Cells of Nicotiana tabacum Grown Under Different Conditions

The root cortex cells of the control *Nicotiana tabacum* plants differ significantly from the cells of plants grown in the presence of peptides (Figure 2). In the control cells, the nuclear chromatin is more decompacted (Figure 2A,B) compared to the chromatin of the nuclei of the experimental samples (Figure 2C–F), in which large chromocenters of heterochromatin are visible in the nuclei. In contrast to control cells, where numerous lytic vacuoles of varying sizes were detected in the cells of the inner (Figure 2A) and outer cortex (Figure 2B), protein-containing vacuoles predominated in the cytoplasm of plant cells in the presence of peptides. Moreover, typical amyloplasts (Figure 2C,B) appeared in the cytoplasm of cells in the presence of AEDL, and proteinoplasts (Figure 2E,F) appeared in the presence of AED(OMe)L, whereas only leukoplasts were present in control cells.

### 2.3. Relative Expression of FREE1 Gene

The FREE1 complex is known to play a key role in PSV biogenesis and the transport of proteins stored in vacuoles [3,4]. FREE1 is required for the formation of a large central lytic vacuole in plant cells. In FREE1 deficiency, the central lytic vacuole is transformed into multiple small vacuoles and leads to the accumulation of a large number of autophagosomes [4].

According to PCR data (Figure 3A), relative *FREE1* gene expression was significantly reduced in *Nicotiana tabacum* roots grown in the presence of AEDL (3-fold) compared to the control. Although *FREE1* gene expression in *Nicotiana tabacum* roots grown in the presence of AED(OMe)L was higher than in the presence of AEDL (1.9-fold), its level remained 1.6-fold lower than in the control.

### 2.4. Analysis Ultrastructure of Vacuoles in the Roots of Nicotiana tabacum Grown Under Different Conditions

Previously, when studying the ultrastructure of *Nicotiana tabacum* root tip cells, we found that plants grown in the presence of the AEDL peptide exhibited numerous large protein storage vacuoles, with large lytic vacuoles observed only in the outer cortex. In contrast, control root cells exhibited only very small and medium-sized lytic vacuoles [14].

Vacuoles found in the cytoplasm of plant cells grown in the presence of peptides differ from control cells in size and the structure of proteins within the vacuoles (Figure 4). In control cells, small vacuoles are present in the cytoplasm of the inner cortex, while larger ones are found in the cells of the outer cortex (Figure 4A,B). In the cytoplasm of the inner cortex cells of roots of plants grown in the presence of AEDL, only protein-containing vacuoles are found (Figure 4D), while large lytic vacuoles are found in the cells of the outer cortex (Figure 4C). In the cells of plants grown in the presence of AED(OMe)L, numerous proteins and protein aggregates are found in the vacuoles, and individual protein bodies are found in the cytoplasm (Figure 4E,F).

### 2.5. Relative Expression of ATG Genes

#### 2.5.1. Formation of Phagophores

In *Nicotiana tabacum* root cells grown in the presence of the AEDL and AED(OMe)L peptides, a 1.49- and 1.56-fold decrease in Atg9 gene expression, respectively, was observed compared to the control (Figure 5A). The decrease in Atg9 transcriptional activity in the presence of the peptides is likely due to excess nutrients accumulated by the cells, while the decrease in membrane transport may lead to the formation of smaller autophagosomes.

#### 2.5.2. Initiation of Autophagosome Formation

We noted an increase in *TOR* expression activity in *Nicotiana tabacum* roots in the presence of AEDL and AED(OMe)L peptides compared to the control by 1.17 and 1.43 times, respectively (Figure 6). The target of rapamycin is phosphatidylinositol 3-kinase-like kinase (PI3K), which functions as a Ser/Thr protein kinase and is a negative regulator of autophagy [17]. PI3K is involved in the phosphorylation of ATG13 [18]. TOR negatively regulates autophagy under nutrient excess by activating the phosphorylation reaction of ATG13, thereby reducing its affinity for ATG1 and decreasing autophagic activity. A significant increase in the relative expression of *PI3K* in *Nicotiana tabacum* roots in the presence of AEDL and AED(OMe)L peptides was noted compared to the control by 3.3 and 4 times, respectively (Figure 6A,B).

#### 2.5.3. Relative Expression of ATG8 Gene

The presence of AEDL and AED(OMe)L peptides in the nutrient medium during *Nicotiana tabacum* cultivation resulted in a 1.68- and 1.37-fold decrease in ATG8 gene expression, respectively (Figure 7). The decrease in ATG8 activity may be due to either a decrease in the number of autophagosomes or a decrease in their size and the accumulation of the ATG8-PE adduct in the cytoplasm.

Figure 7B illustrates the central role of the ubiquitin-linked protein ATG8 in autophagy. During autophagosome formation, ATG8 conjugates with phosphatidylethanolamine (PE).

### 2.6. Immunodetection ATG8

Autophagy is the process of degradation and utilization of damaged molecules and organelles, essential for normal plant development. The autophagic protein ATG8 is conserved across all eukaryotic cells—yeast, animals, and plants. To identify all autophagic structures, we performed immunodetection of the key autophagic protein ATG8 in macerated, detached *Nicotiana tabacum* root tip cells with disrupted cell walls.

ATG8 labels the surfaces of autophagic structures in the cytoplasm of root tip cells *Nicotiana tabacum*. In control cells, the surfaces of autophagosomes localized around the nucleus are intensively labeled (Figure 8, A’’’, B’’’). In the cells of plants grown in the presence of AEDL, autophagosomes labeled with AEDL are also localized near the surface of the nucleus (Figure 8, C’’’, D’’’, D”“’, D”““’, D”“““). Unlike the control and the cells of plants grown in the presence of AEDL, ATG8 is detected in the cytoplasm and as individual punctate sites on the surface of autophagosomes of tobacco cells grown in the presence of AE(OMe)L (Figure 8, E’’’, F’’’).

### 2.7. Analysis Ultrastructure of Phagophores and Autophagosomes

In the cytoplasm of control plant cells, various autophagosomes and vacuoles were found. In plant cells grown in the presence of AEDL, complex autophagic structures were detected (Figure 9D). In the cytoplasm of control plant cells, various autophagosomes and vacuoles are found. In plant cells grown in the presence of AED(OMe)L, numerous phagophores are formed (Figure 9E,F), capturing individual areas of the cytoplasm. In other cells, autophagosomes with various contents formed (Figure 9). We identified cells with examples of typical peroxiophagy, where peroxisomes undergo autophagic degradation (Figure 9H).

### 2.8. Plastid Transformation in Nicotiana tabacum Roots Grown Under Different Conditions

In the cortex cells of the root tip of control plants, typical leucoplasts with a dark stroma and individual lamellae were always detected (Figure 10A,B). Such leucoplasts were present in the cells of the inner root cortex of plants grown in the presence of AEDL (Figure 10C). In contrast, in the cells of the outer cortex, typical amyloplasts with numerous starch granules in the stroma were formed (Figure 10D). Interestingly, in the presence of AED(OMe)L, only typical proteoplasts were detected (Figure 10A,B).

## 3. Discussion

In recent years, interest in the role of short peptides in many cellular processes in plant cells has increased significantly. They have been established as important signaling molecules for intercellular communication. Exogenous peptides AEDL and AED(OMe)L at a concentration of 10^−7^ M act as peptide hormones and stimulate the development of *Nicotiana tabacum* (Figure 1).

The ultrastructure of *Nicotiana tabacum* root cells grown in the presence of exogenous short peptides AEDL and AED(OMe)L was studied for the first time. Differences in the structure of vacuoles, autophagosomes, and leukoplasts were revealed in the inner and outer cortex cells of all studied plants. Larger vacuoles were always present in the outer cortex cells of the control plants (Figure 2A,B; Figure 4A,B). In the inner cortex, only large protein-containing vacuoles were observed in the presence of AEDL (Figure 4C,D) and large vacuoles with proteins and large protein aggregates in the presence of the modified AED(AOMe)L peptide (Figure 4E,F).

To identify autophagic structures in root cells of control plants and plants grown in the presence of peptides, we performed immunodetection of structures labeled with the ATG8 protein. In the presence of exogenous peptides, large autophagosomes and numerous autophagosomes appeared around the nucleus. Small autophagosomes labeled with ATG8 were localized around the nucleus (Figure 8(A’’’–F’’’)), and discrete ATG8 punctate loci marked the membrane surfaces of large autophagosomes (Figure 8(E’’’–F’’’)).

We previously described in detail the autophagic structures formed in the cell cytoplasm in the presence of AEDL (Figure 9C,D) [14].

In the presence of AED(OMe)L, the ultrastructure of autophagosomes changes significantly: they lose their oval shape and are often formed from multiple phagophores (Figure 9E–G). For the first time, a phagosome engulfing peroxisima was detected, indicating the detection of a variant of megaphagy—“pexaphagy”—in the presence of the modified peptide.

Loss of FREE1 function has been shown to result in severe impairments in vacuolar protein transport, vacuole biogenesis, and autophagosome degradation within vacuoles [4]. In tobacco roots grown in the presence of AEDL and its analog AED(OMe)L peptides, inhibition of *FREE1* gene expression is observed. Impairments in the formation and fusion of vacuole and autophagosomal membranes in root cells could be expected. Furthermore, it has been suggested that FREE1, through its ability to bind the phospholipid PI3P, is capable of coordinating the unique plant PI3K complex, regulating autophagosome expansion or maturation [19]. Indeed, the presence of peptides promotes the formation of a large number of small autophagosomes in *Nicotiana tabacum* root cells. Reduced expression of the *ATG9* gene, the only membrane protein in the ATG family involved in PE delivery to phagophore membranes, its expansion, and the formation of autophagosomes, also confirms the formation of small autophagosomes.

Thus, a complex system regulates autophagosome formation and autophagosome degradation. Furthermore, it was noted that FREE1 deficiency is also accompanied by a decrease in vacuole formation and fusion.

Carbon and nitrogen are essential nutrients for cellular function, from which plants synthesize carbohydrates such as sucrose and glucose, which serve as the primary energy source, are structural elements, and utilize ammonia during amino acid biosynthesis [20,21]. Autophagy is one of the mechanisms that ensures nutrient recycling [22]. The autophagosome is a double-membrane structure, which degrades cytosolic components, including damaged organelles (mitochondria, endoplasmic reticulum) and degraded proteins. Mature autophagosomes fuse with vacuoles, where their contents are processed into molecules.

Vacuoles play a key role in maintaining cellular homeostasis and recycling damaged components. Two types of functionally distinct vacuoles can exist within a single cell: the lytic vacuole (LV) and the protein storage vacuole (PSV) [1,2]. LVs contain hydrolytic enzymes for the degradation of damaged and waste material, while PSVs accumulate a large number of different proteins.

Leucine and glutamine are believed to be involved in numerous cell signaling pathways, including inducing autophagy in the event of any deficiency [23,24].

Leucine is an essential amino acid required for cell survival and is involved in the citric acid cycle. Glutamine, although not an essential amino acid, is also involved in the citric acid cycle. It has been suggested that deficiency of these amino acids inactivates TOR, leading to activation of autophagy [25]. The exogenous peptides AEDL and AED(OMe)L that we used at a concentration of 10^−7^ M, containing leucine and glutamic acid, can act as signaling molecules in plant cells.

In recent years, the role of TOR as a global regulator of plant root system development has been actively studied [26]. It has been shown that TOR activation depends on sugars supplied by leaves [27]. The accumulation of carbohydrates in roots is accompanied by metabolic changes in starch, promoting root growth and development [28,29,30,31].

As shown above, in the presence of the exogenous AED(OMe)L peptide, plants have a more developed root system (Figure 1). Furthermore, proteinoplasts were detected in the root cortex cells, which were not formed in the cells of control plants. This reflects increased metabolism and leads to changes in the structure of root cortex leucoplasts (Figure 10E,F). Based on these observations, we hypothesize that the peptide binds to TOR kinase, which is accompanied by the activation of protein kinase S6K and increased protein biosynthesis.

We found that the presence of an exogenous high-negative peptide, AEDL, which binds to TOR kinase, increases its activity and promotes the accumulation of numerous starch granules in the plastids of the root cortex of *Nicotiana tabacum* (Figure 10C,D) and leads to enhanced development of lateral roots (Figure 1).

Based on literary data and our own, a hypothetical scheme for the regulation of primary and lateral root development by the AEDL and AED(OMe)L peptides via binding to TOR kinase was proposed (Figure 11 and Figure 12).

## 4. Materials and Methods

### 4.1. Plant Material

Tobacco plants (*Nicotiana tabacum* L.) of the Samsun variety were grown from seeds in sterile culture in test tubes containing hormone-free Murashige–Skoog (MS) medium. AEDL or AED(OMe)L was added to the medium at a concentration of 10^−7^ M. The plants were grown in a climate chamber with a daily rhythm of 16 h of light and 8 h of darkness. Daytime conditions: 26 °C, 75% humidity; nighttime conditions: 22 °C, 70% humidity. The experiment was carried out for 4 weeks in 4 replicates. The peptides AlaGluAspLeu and AlaGluAsp(OMe)Leu were synthesized by IQChem (St. Petersburg, Russia).

### 4.2. Light Microscopy

Root tips (5 mm) were fixed in 4% paraformaldehyde solution (Sigma Aldrich, St. Louis, MO, USA) in PHEM buffer, pH 6.9 (60 mM PIPES (Sigma Aldrich, St. Louis, MO, USA), 25 mM HEPES (Sigma Aldrich, St. Louis, MO, USA), 10 mM MEGTA (Sigma Aldrich, St. Louis, MO, USA) and 2 mM MgCl2 (Sigma Aldrich, St. Louis, MO, USA) for 1.5 h at room temperature. Then, they were washed with PHEM buffer. Samples were incubated at 7 min in 0.4 M mannitol with 4% cellulase (Sigma Aldrich, St. Louis, MO, USA) and 5 mM EGTA, washed with PBS buffer. Individual cells were obtained from the roots on coverslips. The preparations were dried at +4 °C for 24 h.

### 4.3. Immunodetection of ATG8 Antibodies

The samples were dipped in PBS for 5 min, then in 0.5% Triton X-100 in PBS for 30 min, washed in PBS and incubated for 16–18 h at +4 °C with mouse monoclonal antibodies LC3B Ab-AF4650 (diluted 1:100 in PBS + 0.1% BSA). The preparations were washed and incubated for 45 min at 37 °C with antibodies (diluted 1:50 in PBS + 0.1% BSA) against rabbit IgG conjugated with Texas Red fluorochrome (Sigma Aldrich, USA) used as secondary antibodies; the preparations were washed, stained with DAPI and mounted in Mowiol U-88 (Hoechst, Frankfurt am Main, Germany). The preparations were analyzed under an Olympus BX51 fluorescence microscope (Olympus, Tokyo, Japan), ×100. Images were obtained using a Color View digital camera and Cell software, version 5.1 (Olympus Soft Imaging Solutions, Münster, Germany).

### 4.4. TEM Analysis

Root tips (4–5 mm) were fixed for 24 h in a 2.5% glutaraldehyde solution (Merck, Darmstadt, Germany) in 0.1 M Sorensen’s phosphate buffer (pH 7.2) with 1.5% sucrose. Specimens were washed, postfixed in 1% OsO4 (Sigma-Aldrich, St. Louis, MO, USA), dehydrated in ethanol (30, 50, 70, 96, and 100%) and propylene oxide, and embedded in Epon-812 and Araldite epoxy resins (Merck, Darmstadt, Germany). For electron microscopy, specimens were sectioned on an LKB-V ultramicrotome (LKB, Bromma, Sweden), mounted on formvar-coated grids, and stained with uranyl acetate and lead citrate. Ultrathin sections were then analyzed and photographed using an electron microscope.

### 4.5. Total RNA Isolation and Gene Expression Analysis

The Extran RNA Syntol kit (Syntol, Moscow, Russia) was used for RNA extraction. RNA was isolated from N. tabacum roots. cDNA was synthesized using reverse transcription. cDNA concentration was determined spectroscopically using an IMPLEN nanophotometer. RT-PCR with SYBR Green I was performed on a CFX 96 thermal cycler (BioRad, Hercules, CA, USA). The PCR-RT reaction was carried out under identical conditions for all samples: 95–5 min, then 45 cycles–94–30 s, 58–30 s, 72–30 s. The reaction was carried out in 2–3 parallels and in three repetitions.

Information on the primary structure of *Nicotiana tabacum* genes was obtained from the National Center for Biotechnology Information (NCBI). Primers for these genes were designed using the NCBI Primer-BLAST web tool (https://www.ncbi.nlm.nih.gov/tools/primer-blast/, accessed on 26 May 2026), and synthesized at Syntol (Table 2).

### 4.6. Statistical Analysis

One-way analysis of variance (ANOVA) and Student’s *t*-test (R version 4.3.1) were used for statistical data processing; statistically significant differences were defined at *p* < 0.05. The least significant difference method was used for data processing. Values are presented as mean ± standard deviations for three biological replicates.

## 5. Conclusions

Our study confirmed the role of exogenous peptides in plant development. Differences in the organelle structure of *Nicotiana tabacum* root cells in the control and in the presence of the AEDL and AED(OMe)L peptides were identified. In the presence of the peptides, large protein storage vacuoles, autophagosomes, and leukoplasts were detected in the inner cortex cells. In the outer cortex cells, as in the control, large lytic vacuoles were detected. Unlike the control, in the presence of the exogenous AEDL peptide, amyloplasts were formed as a result of signaling changes in metabolism, while the AED(OMe)L peptide initiated the formation of proteinoplasts.

It is hypothesized that AEDL and AED(OMe)L peptides differentially influence the development and architecture of *Nicotiana tabacum* roots through the activation of TOR kinase.

## Figures and Tables

**Figure 1:**
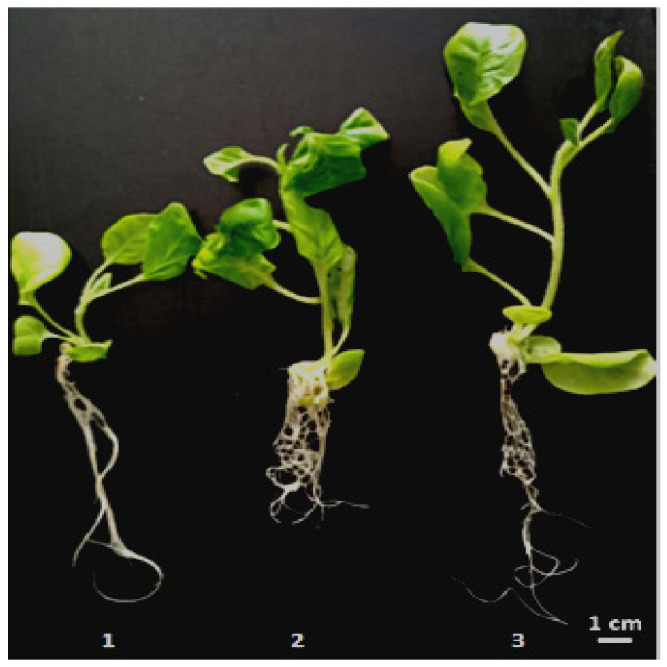
*Nicotiana tabacum*, grown in different conditions. 1—control growth conditions, 2—grown in the presence of AEDL, 3—grown in the presence of AED(OMe)L.

**Figure 2:**
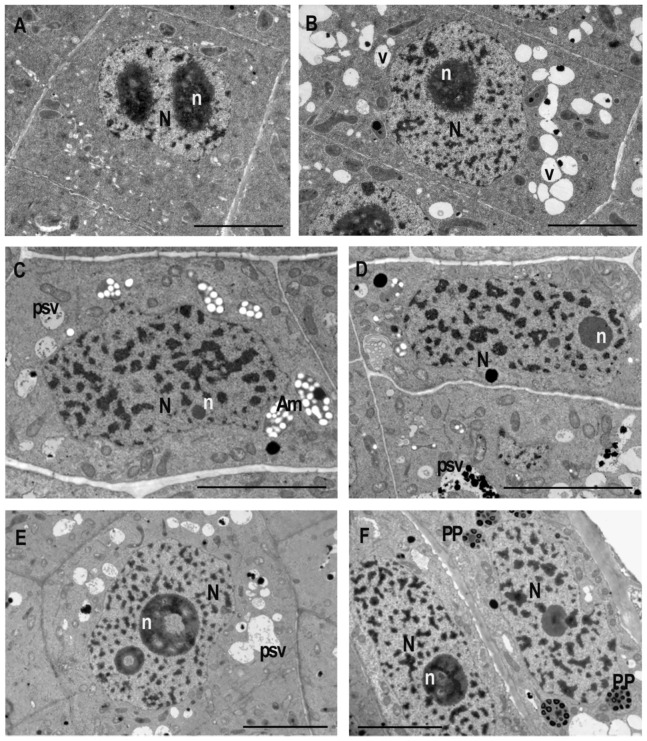
Ultrastructure of inner and outer cortex cells of *Nicotiana tobacum* root tips. (**A**,**B**)—Root cells of control plants, in the cytoplasm of which lytic vacuoles of various sizes and shapes are visible. (**C**,**D**)—Root cortex cells of tobacco grown in the presence of the short peptide AEDL. (**E**,**F**)—Root cortex cells of tobacco grown in the presence of the short peptide AED(OMe)L. N—nucleus; n—nucleolus; V—lytic vacuoles; psv—protein storage vacuoles; Am—amyloplasts; PP—proteinoplasts. Scale bar, 5 μm.

**Figure 3:**
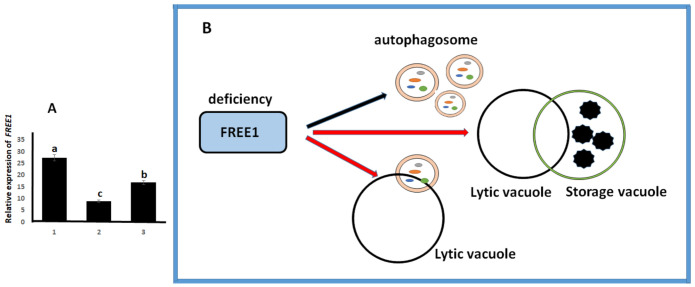
FREE1 influence into formation vacuoles and autopagosomes. (**A**)—Relative expression of *FREE1* gene. 1—control *Nicotiana tabacum*; 2—*N. tabacum* grown in the presence of AEDL; 3—*N. tabacum* grown in the presence of AED(OMe)L. a–c—indicate significant difference using Student’s *t*-test (*p*< 0.05). (**B**)—Schematic representation of the effect of FREE1 deficiency on the inhibition (red arrows) of lytic and storage vacuole fusion, vacuole and autophagosome fusion, and the increase in autophagosome numbers (black arrows).

**Figure 4:**
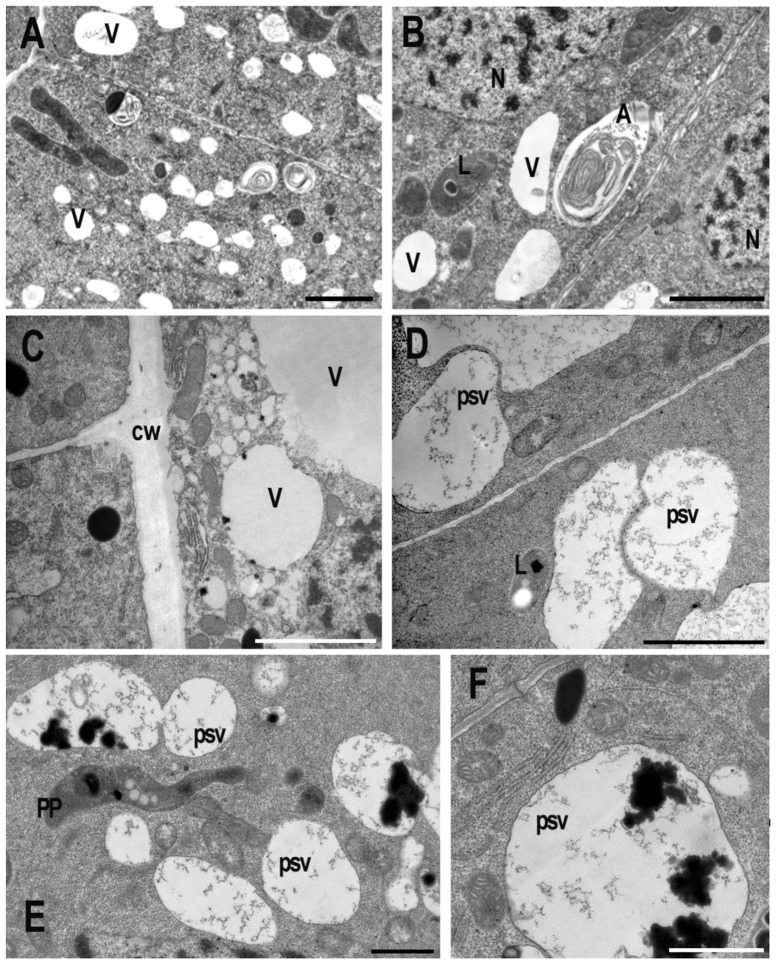
Ultrastructure vacuoles of inner and outer cortex cells of *Nicotiana tobacum* root tips. (**A**,**B**)—Lytic vacuoles of various sizes are present in the cytoplasm of root cells of control plants. Scale bar, 2 μm. (**C**,**D**)—Various storage vacuoles and large lytic vacuoles are detected in the cytoplasm of root cortex cells of tobacco plants grown in the presence of the short peptide AEDL. Scale bar, 2 μm. (**E**,**F**)—Large protein storage vacuoles are detected in the cytoplasm of root cortex cells of tobacco plants grown in the presence of the short peptide AED(OMe)L. Scale bar, 1 μm. N—nucleus; V—lytic vacuoles; A—autophagosome; psv—protein storage vacuoles; PP—proteinoplasts; L—leucoplast; CW—cell wall.

**Figure 5:**
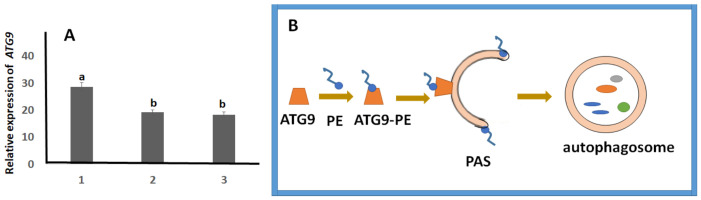
The role of ATG9 in the formation of phagophore membranes. (**A**)—Relative expression of the *ATG9* gene. 1—control *N. tabacum*; 2—*N. tabacum* grown in the presence of AEDL; 3—*N. tabacum* grown in the presence of AED(OMe)L. a–b—indicate significant differences according to Student’s *t*-test (*p* < 0.05). (**B**)—Scheme of formation of the ATG9 complex with PE and incorporation of ATG9-PE into the phagophore membrane, expansion of the phagophore to form an autophagosome. PAS—phagophore structure.

**Figure 6:**
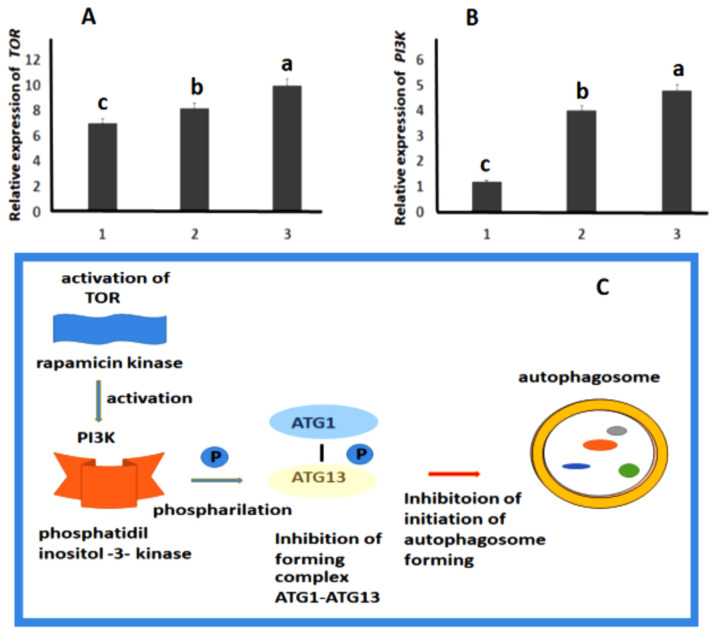
The role of TOR in autophagosome formation. (**A**,**B**)—Relative expression of the *TOR* and *PI3K* genes. 1—control *N. tabacum* sample; 2—*N. tabacum* grown in the presence of AEDL; 3—*N. tabacum* grown in the presence of AED(OMe)L. a–c—indicate significant differences according to Student’s *t*-test (*p*< 0.05). (**C**)—Scheme of initiation of autophagosome formation. TOR regulates PI3K activity, which, through phosphorylation of ATG13, regulates the formation of the ATG13-ATG1 complex, which initiates autophagosome formation.

**Figure 7:**
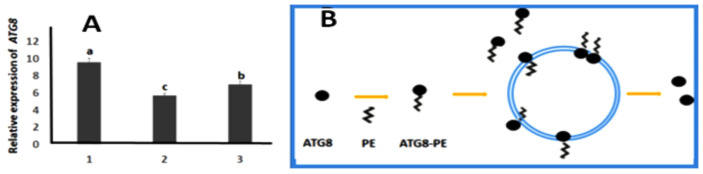
The role of ATG8 in autophagosome formation. (**A**)—Relative expression of the *ATG8* gene. 1—control *N. tabacum*; 2—*N. tabacum* grown in the presence of AEDL; 3—*N. tabacum* grown in the presence of AED(OMe)L. a–c—indicate significant differences according to Student’s *t*-test (*p*< 0.05). (**B**)—Scheme of formation of the ATG8 complex with PE and incorporation of the ATG8-PE complex into the autophagosome membrane and distribution of ATG8-PE on the outer and inner surfaces.

**Figure 8:**
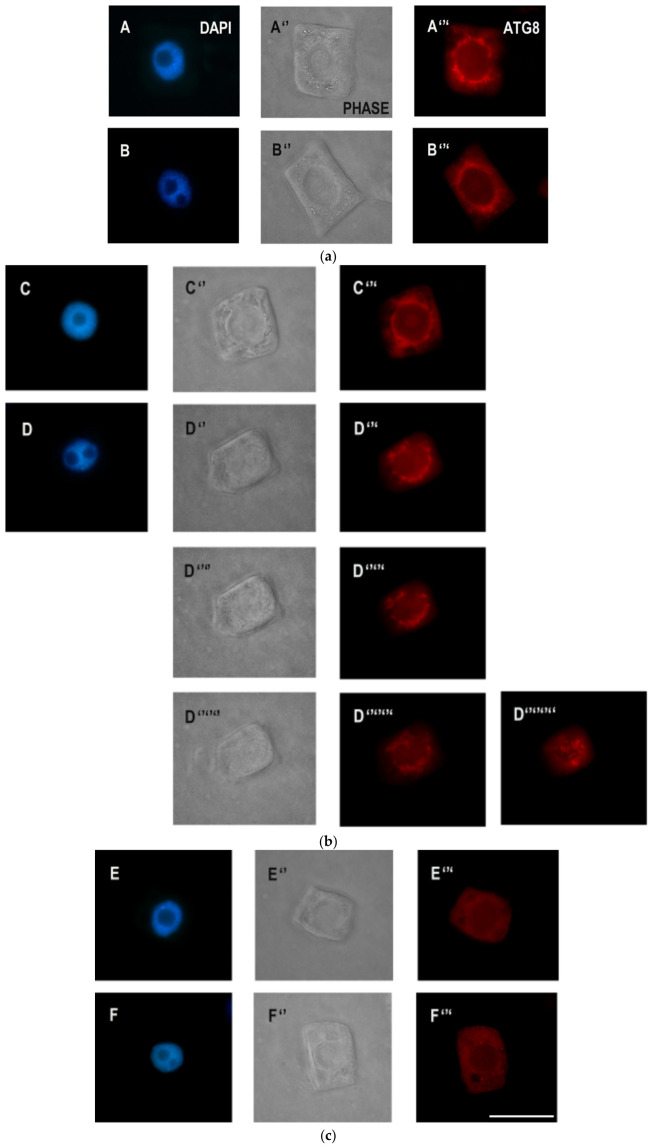
(**a**) Immunodetection of ATG8 on the surface of membranes of autophagic structures of *Nicotiana tabacum* root tip cells. A, B—DNA of the nuclei of root cells of the control plant; A’’, B’’ phase-contrast image of cells; A’’’, B’’’—localization of ATG8 in the cytoplasm of cells. Bar 100 μm. (**b**) Immunodetection of ATG8 on the surface of membranes of autophagic structures of *Nicotiana tabacum* root tip cells. C, D—DNA of the nuclei root cells of plants grown in the presence of ADEL; C’’, D’’, D’“’, D”“’—phase-contrast image of cells in different optical planes; C’’’, D’’’, D”“’, D”““’, D”“““—localization of ATG8 in the cytoplasm of cells in different optical planes. Bar 100 μm. (**c**) Immunodetection of ATG8 on the surface of membranes of autophagic structures of *Nicotiana tabacum* root tip cells. E, F—DNA of the nuclei of root cells of plants grown in the presence of AD(OMe)L; E’’, F’’—phase-contrast image of cells; E’’’, F’’’—localization of ATG8 in the cytoplasm of cells. Bar 100 μm.

**Figure 9:**
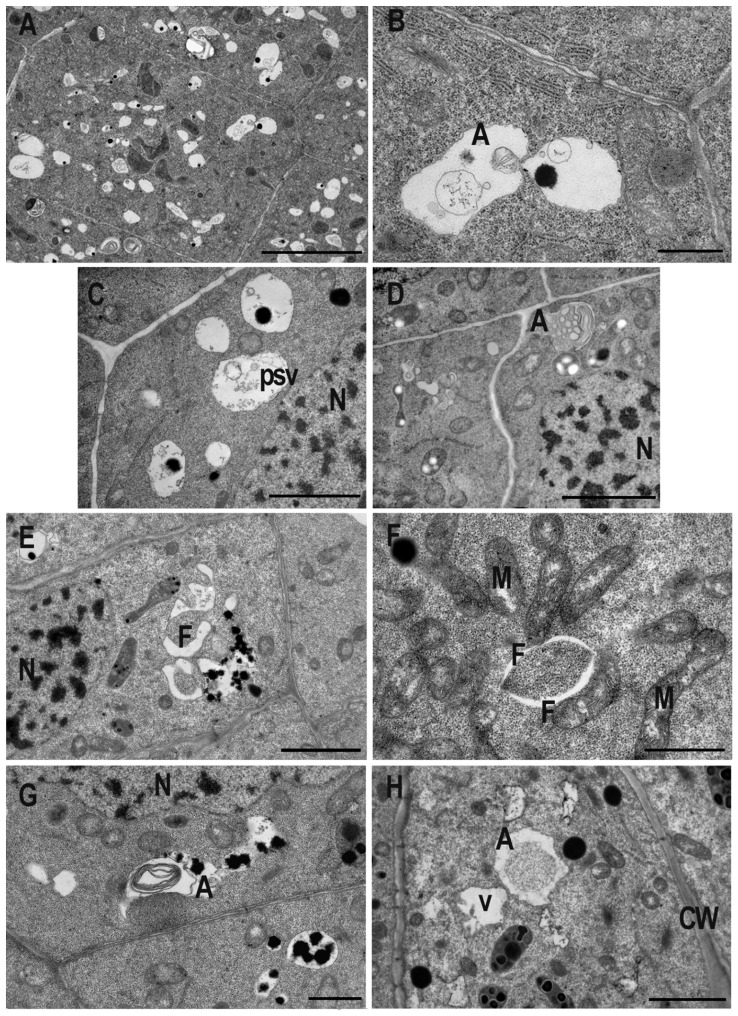
Ultrastructure autophagosomes structures of cortex cells of *Nicotiana tobacum* root tips. (**A**)—Autophagosome structures of various sizes are present in the cytoplasm of root cells of control plants. Scale bar, 5 μm. (**B**)—Enlarged area of cytoplasm with autophagic structures. Scale bar, 1 μm. (**C**,**D**)—Autophagic structures in the cytoplasm of plants grown in the presence of AEDL. Scale bar, 2 μm. (**E**,**F**)—Autophagic structures (phagophores) in the cytoplasm of plant root cells grown in the presence of AED(OMe)L. Scale bar, 2 μm (**E**) and 1 μm (**F**). (**G**)—Complex autophagic structure in the cytoplasm of plant root cell and (**H**)—autophagosome engulfing peroxisome in the cytoplasm of plant root cells grown in the presence of AED(OMe)L. Scale bar, 1 μm (**G**) and 2 μm (**H**). N—nucleus; V—lytic vacuoles; psv—protein storage vacuoles; A—autophagosome; F—phagopore; M—mitochondria; CW—cell wall.

**Figure 10:**
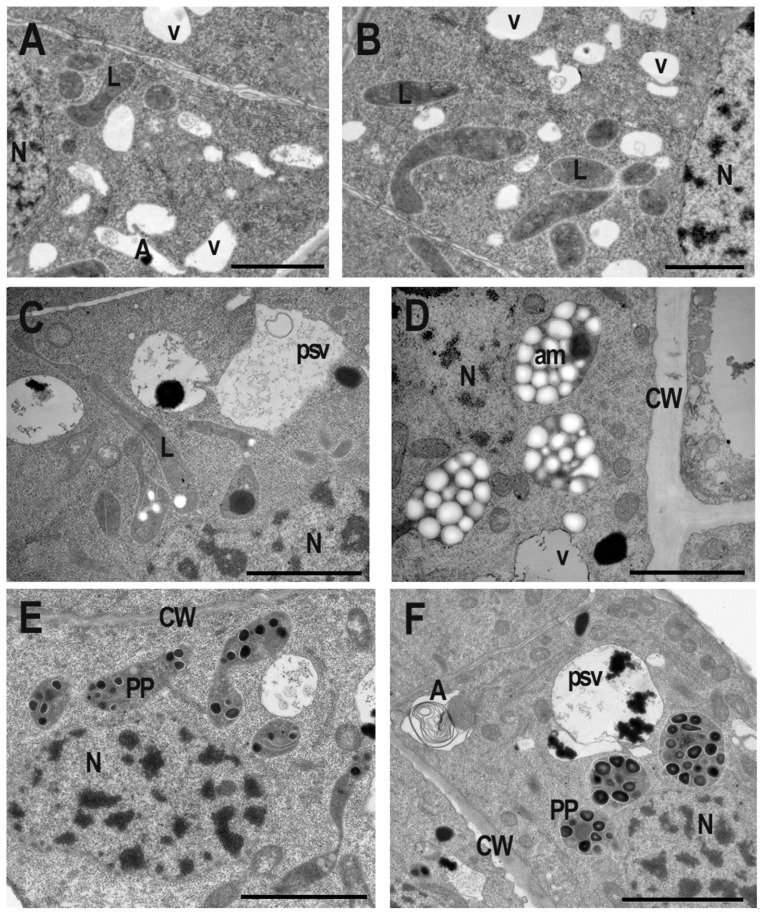
Ultrastructure leucoplasts structures of cortex cells of *Nicotiana tobacum* root tips. (**A**,**B**)—typical leukoplasts of root tip cortex cells. (**C**,**D**)—into the cells of the inner cortex of the root tip of plants grown in the presence of AEDL, only typical leukoplasts are detected, while in the cells of the outer cortex, typical amyloplasts are found. (**E**,**F**)—In the cortex cells of the root tip of plants grown in the presence of AED(OMe)L, only typical proteoplasts are detected. N—nucleus; V—lytic vacuoles; psv—protein storage vacuoles; A—autophagosome; L—leucoplast; am—amyloplast; PP—proteoplast; CW—cell wall. Scale bar, 2 μm.

**Figure 11:**
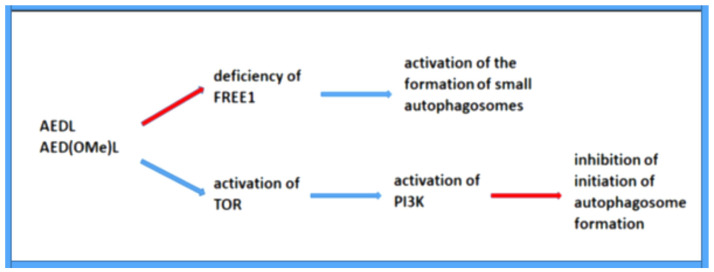
Hypothetical scheme of regulation of formation autophagosomes in roots of *Nicotiana tabacum* by AEDL and AED(OMe)L peptides through inhibition of FREE1 and activation of TOR kinase.

**Figure 12:**
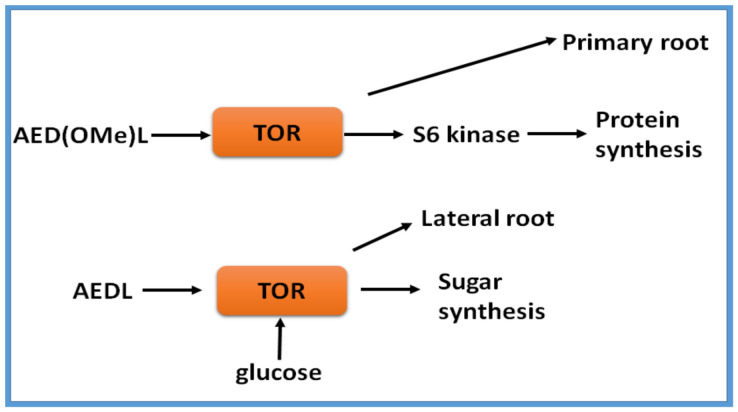
Hypothetical scheme of regulation of development of primary and lateral roots of *Nicotiana tabacum* by AEDL and AED(OMe)L peptides through activation of TOR kinase.

**Table 1:** Morphometric parameters of *Nicotiana tabacum* growth under different conditions.

Variant	Root Length, cm	Shoot Height, cm	Crude Weight, g
Control	9.0 ± 0.45	7.0 ± 0.35	0.53 ± 0.03
+AEDL	8.0 ± 0.36	12.5 ± 0.61	1.59 ± 0.08
+AED(OMe)L	14.6 ± 0.57	13.5 ± 0.66	1.77 ± 0.09

Weight—total weight of root and shoot. Data were expressed as mean ± standard deviation (SD; *n* = 3).

**Table 2:** Primers for RT-PCR.

Gene	5′–3′-Sequence
***ATG9***	tga aca ccc cat gga gag ga
	gga acc caaatg tat tttct
***ATG8e***	ttcttcttcatccaccaccgc
	tgcaaacacagcctacaacta
***PI3K***	cggaaacgagttccgcttttt
	ctctggctttttctcctccgt
***TOR***	ctcgggagg tag aacctg ga
	agcaagctacctgcc tac ct
***FREE1***	ccgatg cat ggcagaaggt aa
	aagaggggg aga aaagcatgg

Data on the *ATG* gene structure in *N. tabacum* were obtained from NCBI. Primers for the genes were synthesized by Syntol. The *GaPDh* gene was used as a reference gene. Each RT-PCR reaction was performed in triplicate.

## Data Availability

The original contributions presented in this study are included in the article. Further inquiries can be directed to the corresponding author.
